# Enhanced humoral response in pregnant mice immunized with liposome encapsulated recombinant neutralizing epitope protein of Hepatitis- E virus

**DOI:** 10.1186/s12985-015-0302-8

**Published:** 2015-05-03

**Authors:** Shivali Shirish Joshi, Vidya Avinash Arankalle

**Affiliations:** National Institute of Virology, 130/1, Sus Road, Pashan, Pune, 411021 India; ICMR consultant, National Institute of Virology, 130/1, Sus Road, Pashan, Pune, 411021 India

## Abstract

**Background:**

Pregnant women from developing countries are at high-risk of hepatitis E-associated high mortality and constitute priority population for vaccination. So far, candidate vaccines have not been evaluated during pregnancy. We evaluated our vaccine candidate, recombinant Neutralizing Epitope protein (rNEp) encapsulated in liposomes, in pregnant mice.

**Methods:**

A single dose (10 μg) of the formulation was administered intramuscularly on day 7 of pregnancy. Titres of serum IgG antibodies to hepatitis E virus (IgG-anti-HEV), levels of cytokines and biochemical parameters were determined. Spleens were harvested from pregnant and non-pregnant mice for immunophenotyping (flow cytometry), cytokines (cytometric bead array, CBA) and gene expression of immune response genes (Taqman low density array, TLDA). Histopathology studies of spleen, liver, kidneys, brain and muscle was carried out.

**Results:**

The vaccine was well-tolerated during pregnancy as evidenced by histopathology and serum biochemical parameters. Anti-HEV titres were significantly higher in the pregnant balb/c and C57BL/6 mice (3592 ± 802 and1016 ± 138 respectively, than in non-pregnant groups (634 ± 191 and 320 ± 55 respectively, p < 0.001 for both) suggesting that the higher antibody response in pregnant mice was independent of the genetic makeup of the host but immunogen-driven. Pups receiving vertically transferred antibodies developed lower anti-HEV antibodies (p < 0.05) when immunized with the formulation after seronegativity than in the age-matched mice without such antibodies. In non-pregnant mice, a Th1 response and discordance between splenic and serum cytokines was evident while in pregnancy, a Th2 bias was observed irrespective of immunization. Increased CD19 levels correlated with higher anti-HEV titres in pregnant mice.

**Conclusion:**

The single dose of the vaccine was safe and highly immunogenic in pregnant mice. Degree and type of immune response to vaccination during pregnancy is immunogen-driven. In-depth studies are needed to understand the underlying immunologic mechanism(s). These encouraging results for a vaccine intended for use in pregnant women should be confirmed in higher animals.

## Background

Hepatitis E is a major public health problem in developing countries and causes waterborne epidemics and sporadic disease. Hepatitis E virus (HEV) has predilection for young adults and causes high mortality (~20%) among pregnant women, especially in the later trimesters [1]. Therefore, pregnant women from endemic countries are considered the ideal category for hepatitis E immunization. So far, 10 vaccine candidates including ours were shown to be efficacious in the preclinical trial in rhesus monkeys [2-11] and two have undergone clinical trials [12,13]. However, except for incidental immunization of pregnant women during a clinical trial [14], none of these were evaluated during pregnancy.

Small laboratory animals are not susceptible to HEV and the virus does not grow to high titres in culture systems, eliminating possibility of traditional live/attenuated vaccines. Development of recombinant vaccines remains the best possible option, with most vaccine efforts focused on the Open Reading Frame-2 (ORF-2) capsid protein. Of the two vaccine candidates completing clinical trials, one was a 56 kDa protein produced in insect cells which showed 95.5% efficacy after administration of three doses of 20 μg each at 0, 1 and 6 months [13]. The other was a bacterially expressed protein HEV239, which showed 100% efficacy on administration of three doses of 30 μg each at 0, 1 and 6 months [12]. This vaccine, Hecolin® is now commercially available for use in China, but not globally, so far. Subsequent use of this vaccine in the community confirmed no adverse effects, protection for 4.5 years and continued monitoring [15]. Cross-protective efficacy was evident as the predominant strain in the area was genotype-4 while the vaccine was derived from genotype-1 virus.

HEV-ORF2 is highly conserved among HEV species and encodes for a single structural protein (660aa, 88 kDa) that has been the target for vaccine development. With the identification of a neutralization epitope (NE, nt458-607, 150aa) within ORF-2 in 2004 [16], we evaluated the utility of this smaller region in vaccine development. It was subsequently shown that the ORF2-encoded protein forms the capsid through its homodimeric subunits (domain E2 amino acids 394–606 and domain E2s amino acids 455–602) that is essential for HEV interaction with the host cell. The neutralizing antibody recognition site of HEV was mapped the on the E2s (I) domain [17].

Our initial studies in mice showed that the NE-based DNA-prime-protein-boost (DPPB) approach was superior to NE-DNA and ORF-2-DPPB formats [18]. ORF-2 and NE regions were further evaluated in rhesus monkeys. Of these, liposome encapsulated DNA and protein formulations as well as NE-DNA-DPPB approach led to sterilizing immunity [8]. When only NE protein similarly encapsulated in liposomes and administered to rhesus monkeys, similar protection was recorded (Arankalle et al., manuscript in preparation). NE protein in combination with hepatitis B surface antigen (HBsAg) was shown to be highly immunogenic to both the components in mice [19] and rhesus monkeys [8].

In the absence of an effective treatment, protection of pregnant women from hepatitis E-associated mortality and morbidity by immunization is an effective alternative approach. Immunization of women in child-bearing age, though possible, is not practical and economically viable in the resource-restricted countries needing hepatitis E vaccine. As pregnant women routinely receive vaccines, targeting this at-risk population appears to be feasible. So far, hepatitis E vaccine has not been assessed during pregnancy. As a first step, we evaluated our vaccine candidate in pregnant mice and report a preliminary analysis of the possible factors responsible for the observed differences in the antibody titres with respect to pregnancy.

## Results

### Preparation of the immunogen

**Figure 1:**
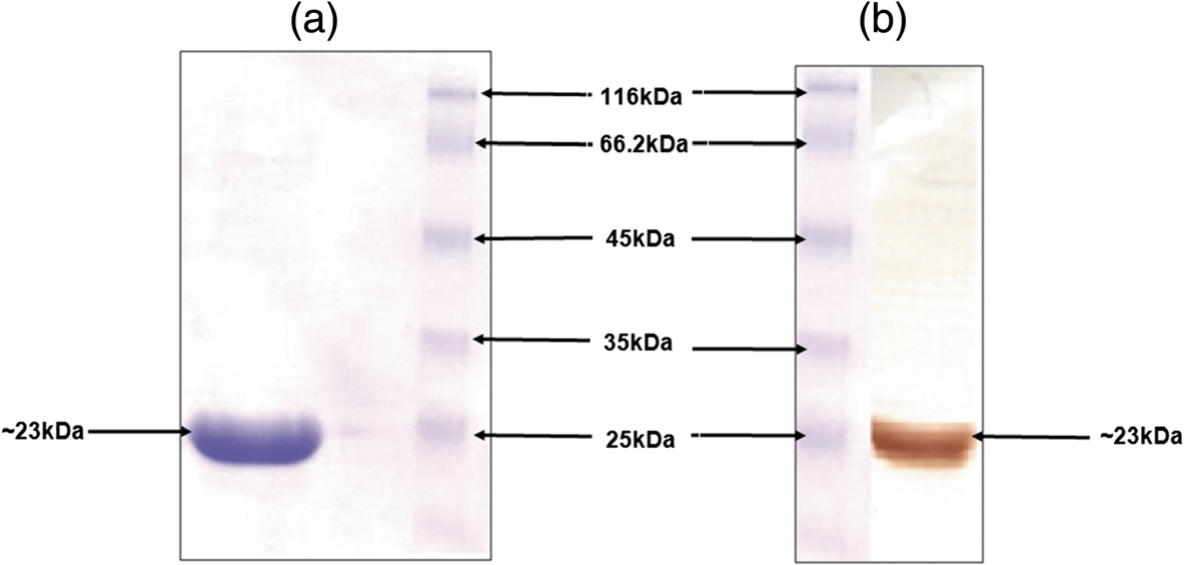
Immunogen preparation **(a)** Depicts purified NE protein of expected size (23 kDa) on 12% SDS-PAGE and **(b)** Immunoreactivity of the protein with IgG anti-HEV positive human serum in western blot.

Figure 1a depicts purified recombinant neutralizing epitope protein (rNEp) as a single band of ~23 kDa with strong immunoreactivity in western blot (Figure 1b). Endotoxin levels were less than 10 EU/mg of protein.

### ELISA

#### Antibody titres in pregnant/non-pregnant mice

**Figure 2:**
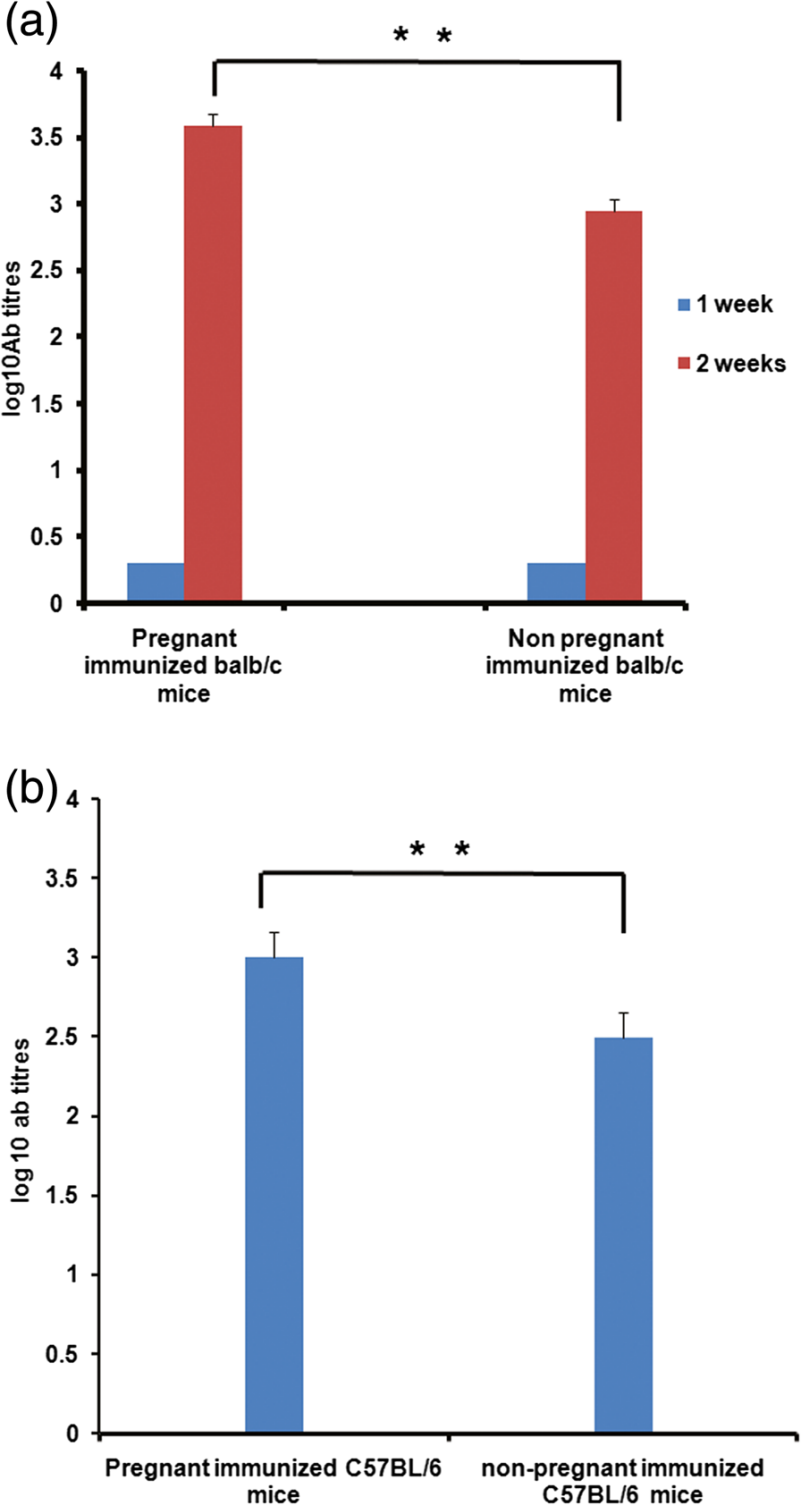
IgG Anti-HEV titres in **(a)** pregnant and non-pregnant balb/c mice at 1 and 2 weeks post dose, **(b)** pregnant and non-pregnant C57BL/6 mice at 2 weeks post dose. **indicates significant difference; p values < 0.001 (t-test).

At one week post-immunization, no seroconversion was noted in either mice group whereas 100% seroconversion was observed 2 weeks post-dose in both pregnant and non-pregnant balb/c mice. Geometric mean titres (GMT) in the pregnant mice (3592 ± 802) were significantly higher (p < 0.001) than in the non-pregnant mice (634 ± 191.3) (Figure 2a). In C57BL/6 mice, 100% seroconversion was observed 2 weeks post-dose in both pregnant and non-pregnant mice. Anti-HEV titres were significantly higher (p < 0.001) in the pregnant mice (1016 ± 138) than in the non-pregnant mice (320 ± 55) (Figure 2b).

#### Antibody titres in immunized-mother-mice and pups

**Figure 3:**
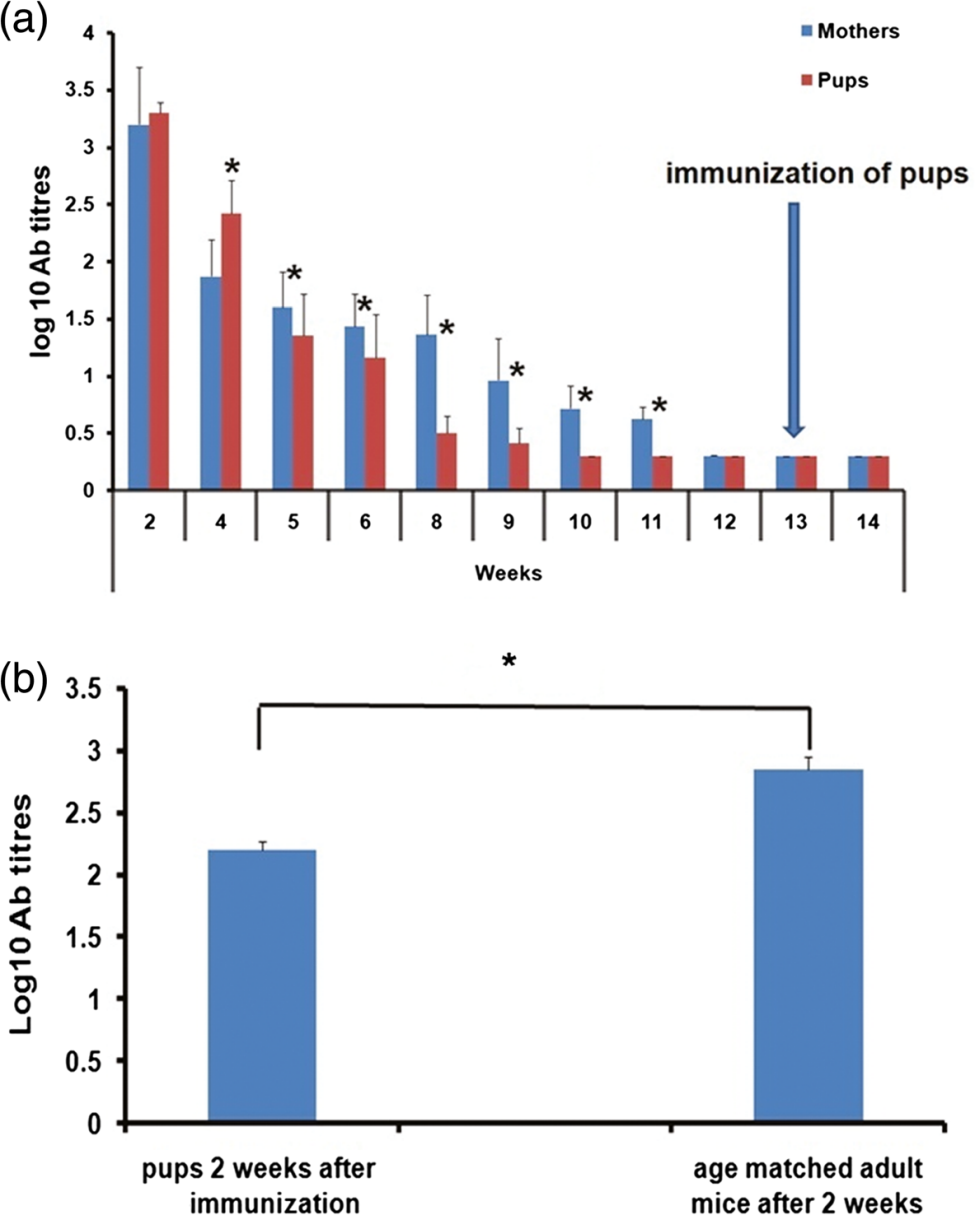
IgG anti-HEV titres in **(a)** balb/c mothers and pups at different intervals, Arrow indicates time for immunization of pups **(b)** in balb/c mice with or without vertically transferred antibodies , 2 weeks post dose. *indicates significant difference , p values < 0.05 (t-test).

IgG anti-HEV antibodies persisted in the mothers till 11 weeks and in the corresponding pups till 9 weeks after birth (Figure 3a). At 4 weeks, anti-HEV titres were higher in the pups than in the mothers (p < 0.05) and declined subsequently. During 5–12 weeks, antibody titres were higher in the mothers (p < 0.05)

#### Anti-HEV response of pups receiving vertically transferred anti-HEV antibodies to active immunization with the candidate vaccine

At 9 weeks after birth, the pups scored IgG anti-HEV negative. When a single dose of the same formulation was administered to these pups at 13 weeks, anti-HEV titers were significantly lower (168 ± 50) than the age matched mice (580 ± 118) receiving the same dose (p < 0.05, Figure 3b)

### Immunophenotyping of splenocytes

**Figure 4:**
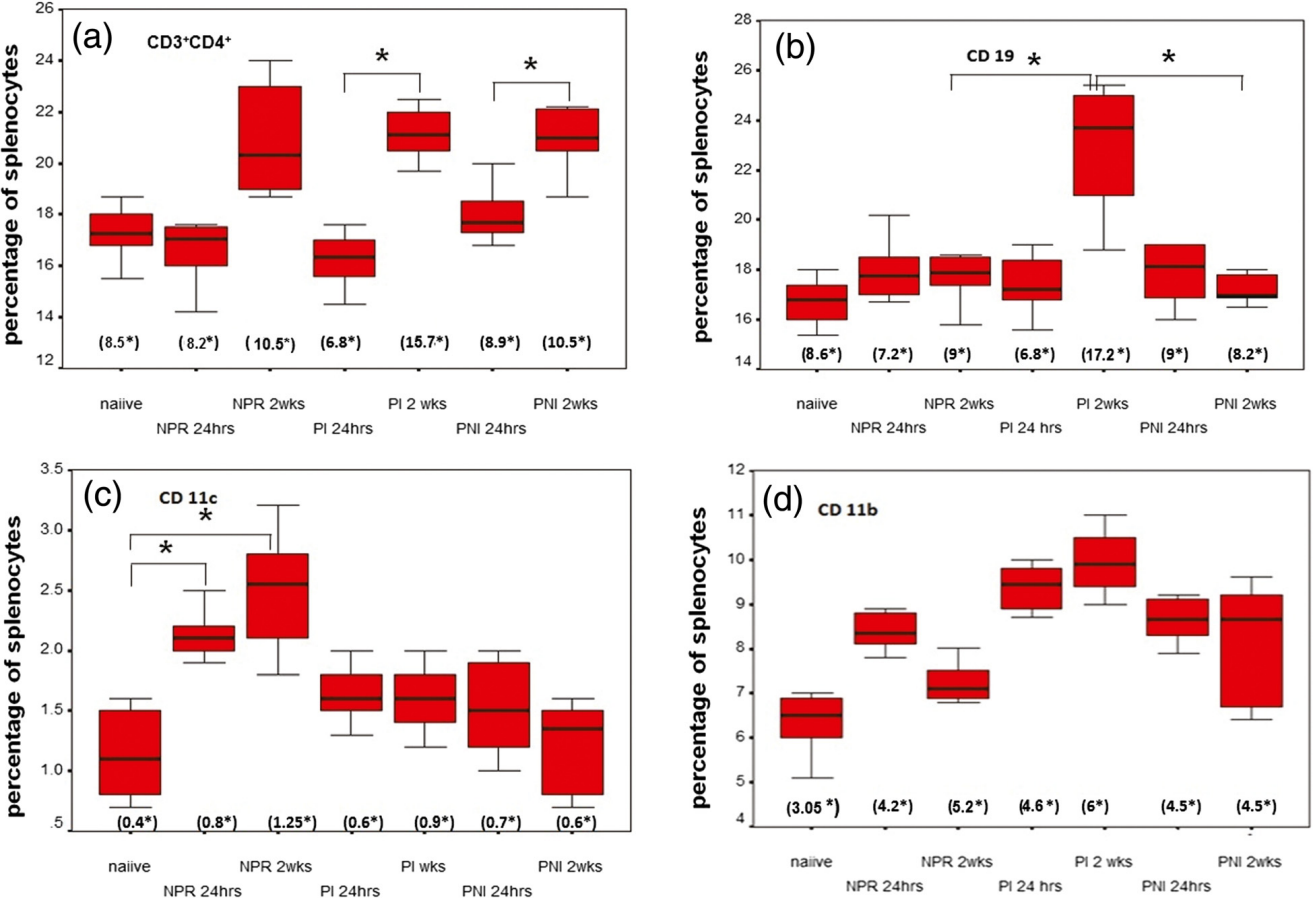
Surface staining of splenocytes of mice groups at different time points. **(a)** CD3^+^CD4^+^ T cells **(b)** CD19^+^ B cells **(c)** CD11c^+^ dendritic cells **(d)** CD11b^+^ macrophages. Values represent as mean percent of gated splenocytes ± S.D. NPR = non – pregnant immunized mice, PI = pregnant immunized mice and PNI = pregnant non-immunized mice. Values in parentheses represent means of absolute counts in spleen. *within the parentheses denotes multiplied by 10^6.^ *indicates p value < 0.05 (ANOVA).

We first assessed the surface markers for immune cell types important in Th1 /Th2 and humoral responses i.e., T helper cells (Figure 4a), B cells (Figure 4b), dendritic cells (Figure 4c) and macrophages (Figure 4d). In non-pregnant mice, a significant rise was noted in the percentage of CD11c^+^ cells at 24 hrs post-dose (p < 0.05).

CD3^+^CD4^+^ cells increased with pregnancy duration (p < 0.05). Though statistically insignificant, the numbers of CD11b^+^ cells were higher in the pregnant mice. Following immunization, CD19^+^ cells increased significantly at 2 weeks post-dose in the pregnant mice when compared to the pregnant controls or non-pregnant-immunized groups (p < 0.001 for both).

The levels of co-stimulatory/activation markers i.e., CD11c^+^CD80^+^, CD11c^+^CD86^+^, CD11c^+^MHC-II^+^, CD19^+^MHCII^+^, CD11b^+^MHCII^+^, CD11c^+^D69^+^, CD19^+^CD69^+^, CD11b^+^CD69^+^, CD4^+^CD69^+^ were independent of pregnancy and immunization (p > 0.05, data not shown). Though higher number of CD19^+^CD69^+^ cells was recorded in the pregnant mice at 24 hrs post-dose, the difference was statistically insignificant.

### Estimation of cytokines in NE-stimulated splenocyte cultures

**Figure 5:**
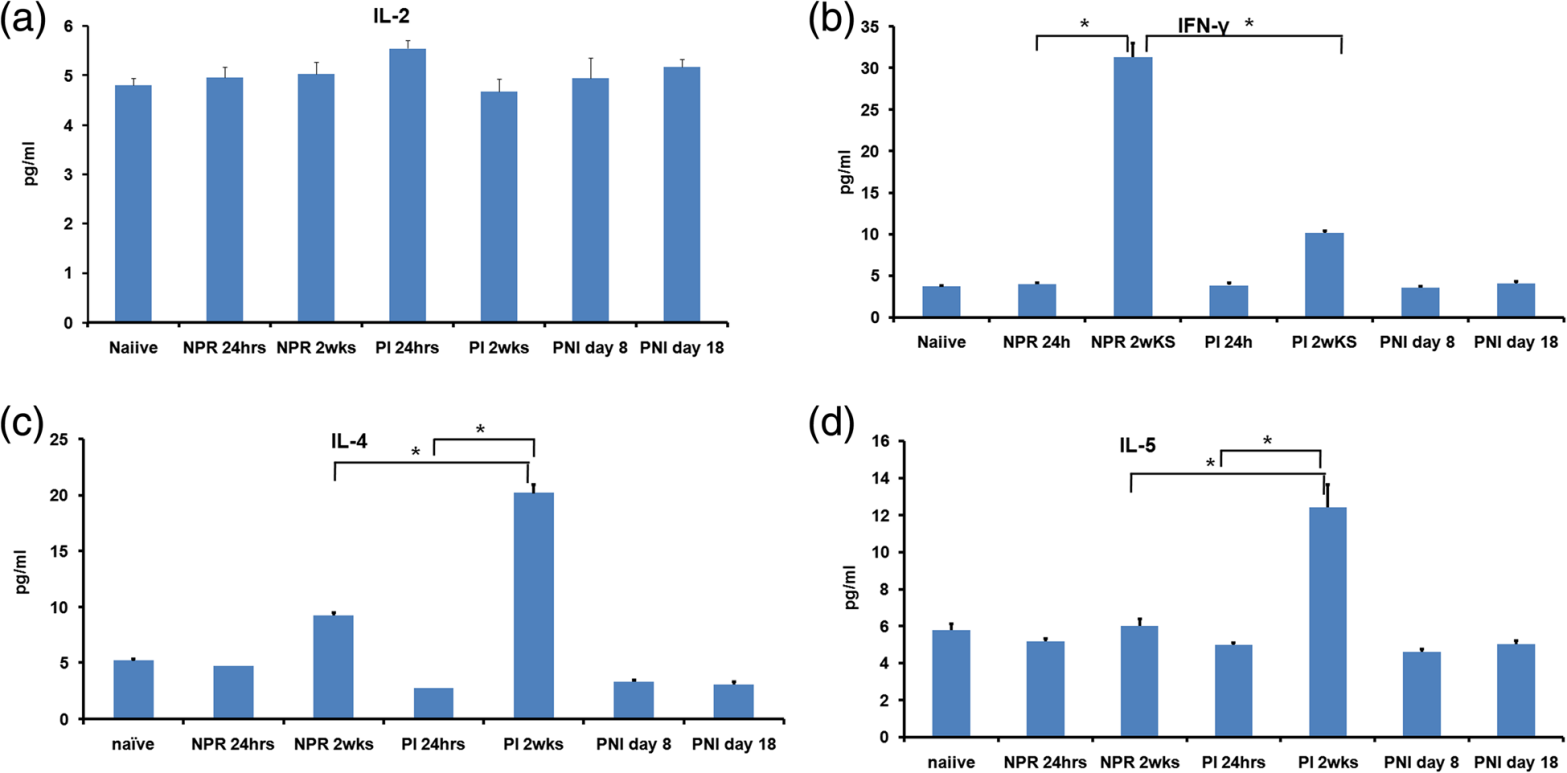
Cytokine levels in pg/ml ± S.E (standard error) in NE-restimulated splenocyte supernatants at 72 hrs of **(a)** IL-2 **(b)** IFN-γ **(c)** IL-4 and **(d)** IL-5. Values represent mean cytokine measurements from different mice belonging to one group NPR = non–pregnant immunized mice, PI = pregnant immunized mice and PNI = pregnant non-immunized mice. *indicates p value <0.05(ANOVA).

Comparisons of NE-specific Th1/Th2 response showed that IL-2 levels remained comparable between non-pregnant and pregnant groups at 24 hrs and 2 weeks (Figure 5a). At 2 weeks post-immunization, IFN-γ levels increased in both non-pregnant (8fold) and pregnant (4fold) mice, when compared to the respective, 24 hrs-post-immunization categories (p < 0.05, Figure 5b). IFN-γ levels at 2 weeks post immunization were significantly lower in the immunized pregnant mice compared to non-pregnant mice (p < 0.05). An increase was noted for IL-4 in non-pregnant (2fold) and pregnant (7.5fold) mice at 2 weeks compared to 24 hrs post dose (p < 0.05, Figure 5c). IL-5 levels were unchanged in non-pregnant mice at both time points, while in the immunized pregnant mice at 2 weeks, a 2.3 fold increase compared to 24 hrs was noted.(p < 0.05) (Figure 5d). Levels of IL-4 and IL-5 in the pregnant immunized group at 2 weeks were significantly higher than that of the non-pregnant mice at 2 weeks. (p < 0.05)

### Estimation of serum cytokines

#### Th1 cytokines

None of the Th1 cytokines (IL-2/IFN-γ/IL-12p40/IL-p70) were detected in the unimmunized and immunized non-pregnant mice. IL-2 levels (22.17 ± 0.21 pg/ml) were higher during early-pregnancy, declining to 4.7 ± 0.2 pg/ml in the later phase (p < 0.001). Immunization did not alter IL-2 levels. The increase in IL-12p40 levels at 2 weeks post-dose (28.9 ± 2.2 pg/ml) was reflection of pregnancy status (19.5 ± 0.40 pg/ml). During early pregnancy, immunization led to significant induction of IL-12p70 (30.16 ± 2.4 pg/ml to 103 ± 7.2 pg/ml, p < 0.001). The levels were comparable at 2 weeks (40.8 ± 3.1, 36.4 ± 1.4 pg/ml respectively). IFN-γ was undetectable during healthy pregnancy, elevated at 24 hrs (56.6 ± 5.7 pg/ml, p < 0.001) and reduced (6.2 ± 1.1 pg/ml, p < 0.001) at 2 weeks post-dose (Table 1).

**Table 1:** **Estimation of serum Th1/Th2 cytokine levels**

**Groups**	**Time point**	**Cytokine levels pg/ml (Std.error)**					
		**IL-2**	**IFN-γ**	**IL-12p40**	**IL-12 p70**	**IL-6**	**IL-10**
Naiive		0.2(0)	0.2(0)	0.2(0)	0.2(0)	0.2(0)	12.0 (2.3)
NPR*	24 hrs	0.2(0)	0.2(0)	0.2(0)	0.2(0)	20.9 (1.92)	4.1 (1.1)
NPR	2 wks	0.2(0)	0.2(0)	0.2(0)	0.2(0)	8.3 (0.2)	10.2 ( 0.1)
PI**	24 hrs	19.4 (1.1)	56.6 (5.8)	0.2(0)	103 (7.2)	45.9 (1.4)	11.3 (1.5)
PI	2wks	2.7 (0.2)	6.2 (1.1)	28.9 (2.2)	36.4 (1.4)	25.5 (0.5)	23 (2.5)
PNI***	Day8	22.2 (0.2)	0.2(0)	0.2(0)	30.2 (2.4)	12.9 (0.7)	15.4 (0.4)
PNI	Day18	4.7 (0.2)	0.2(0)	19.6 (0.4)	40.8 (3.1)	0.2(0)	25.6 (2.5)
p-values							
naiive vs NPR 24 hrs		N.S	N.S	N.S	N.S	<0.001	N.S
naiive vs NPR 2wks		N.S	N.S	N.S	N.S	<0.001	N.S
NPR 24 hrs vs NPR 2wks		N.S	N.S	N.S	N.S	<0.05	<0.05
PI 24 hrs vs PI 2wks		<0.001	<0.001	<0.001	<0.05	N.S	<0.05
PNI day8 vs PNI day18		<0.001	N.S	<0.05	N.S	<0.05	<0.05
PI 24 hrs vs PNI day8		N.S	<0.001	N.S	<0.001	<0.05	N.S
PI 2wks vs PNI day18		N.S	<0.05	N.S	N.S	<0.001	N.S
NPR 24 hrs vs PI 24 hrs		<0.001	N.S	<0.001	<0.001	<0.05	<0.001
NPR 2wks vs PI 2wks		<0.001	<0.001	<0.001	<0.001	<0.001	<0.05

*indicates non-pregnant immunized group, **PI = pregnant immunized group, ***PNI = pregnant non-immunized mice group.

N.S. = not significant, p > 0.05. (ANOVA test).

#### Th2 cytokines

Of the 3 Th2 cytokines estimated (IL-4/IL-6/IL-10), IL-4 was undetectable in all the mice groups. Non-pregnant mice exhibited increased IL-6 levels at 24 hrs (20.9 ± 1.9 pg/ml, p < 0.001) declining to 8.3 ± 0.17 pg/ml at 2 weeks post-dose (p < 0.001). Immunization during pregnancy led to an increase in IL-6 levels (12.9 ± 0.74 to 45.9 ± 1.4 pg/ml, p < 0.005) in the early phase and declined to 25 ± 0.5 pg/ml (p < 0.005) at 2 weeks post-dose. IL6 levels were higher at 2 weeks post-dose than in the respective unimmunized category (p < 0.05). IL-10 levels declined in immunized non-pregnant mice at 24 hrs (4 ± 1 pg/ml) as compared to the controls (12 ± 2.3 pg/ml, p < 0.05), returning to normal levels at 2 weeks (10.2 ± 0.1 pg/ml). Pregnancy led to increased IL-10 levels in the late phase (25.6 ± 2.5 pg/ml) that was not altered by immunization (23 ± 2.5 pg/m, p > 0.05) (Table 1).

#### Th1/ Th2 ratio in pregnancy

In the absence of detectable IFN-γ and IL-4 levels, we compared IL-2/IL-10 ratios. During later pregnancy, levels of the Th1 cytokine (IL-2) decreased from 22.2 to 4.7 while the Th2 cytokine (IL-10) increased from 15.4 to 25.6, the Th1/Th2 ratio decreasing from 1.44 to 0.18, suggestive of Th2 bias. Following immunization, the Th1/Th2 ratio dropped to 0.11 (Table 2).

**Table 2:** **Th1/Th2 ratios in pregnant mice**

**Category**	**Pregnancy duration**	**IL-2 (pg/ml )**	**IL-10 (pg/ml)**	**Ratio (IL-2/IL-10)**
PNI*	8 (early)	22.2	15.4	1.44
PNI	18 (late)	4.7	25.6	0.18
PI**	8 (24 hrs post dose)	19.4	11.3	1.71
PI	18 (2 weeks post dose)	2.7	23	0.11

*is pregnant non-immunized group, **is pregnant immunized group.

### mRNA expression levels of the immune response genes

**Figure 6:**
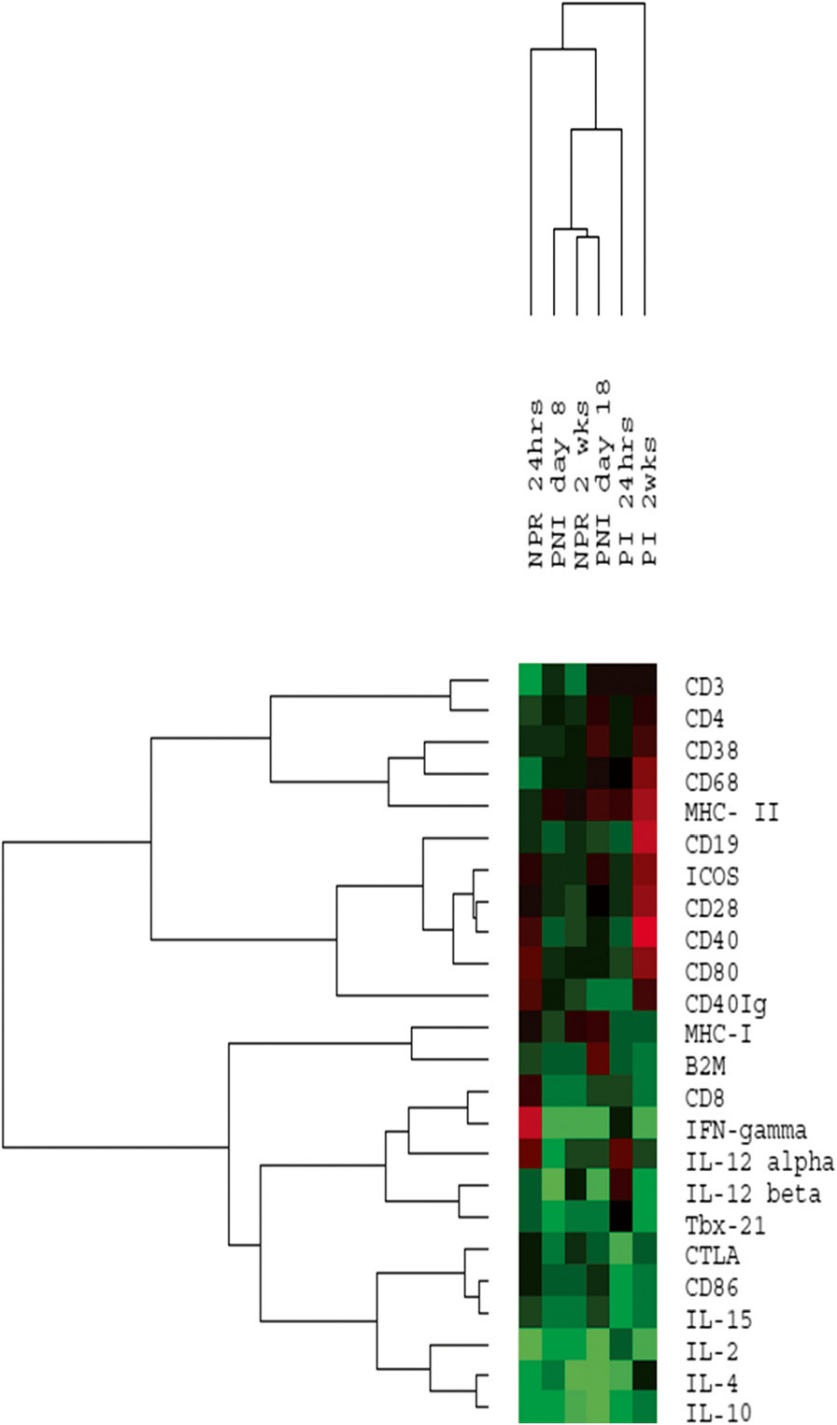
Heatmap of immune response genes in different mice groups. NPR = non-pregnant immunized group, PI = pregnant immunized and PNI = pregnant unimmunized group. Green color indicates downregulation, black represents normal expression and red indicates upregulation of genes.

The heatmap (Figure 6) depicts that the pregnant immunized group at 2 weeks was distinctly different, healthy pregnancy and non-pregnant immunized group at 2 weeks clustered together whereas non-pregnant and pregnant groups at 24 hours were different. In the non-pregnant immunized group, except for the downregulation of CD3 at 24 hrs (2.7fold), all the other T-Cell surface molecule genes were within the normal range at both time points. During normal pregnancy, only CTLA was downregulated (2.1fold) that was similar (3.4fold) at 24 hours-post-dose and normal at 2 weeks. All the B-cell surface marker genes were normally expressed in the non-pregnant mice at both time points post-immunization. Pregnancy did not alter expression of these genes. However, an elevation was seen at 2 weeks-post-dose in CD19 (2.8) and CD40Ig (3.3) levels, normally expressed at 24 hrs. The other cell-surface-markers were within the normal range in the immunized non-pregnant and pregnant unimmunized mice groups. In the immunized pregnant group, upregulation of CD80 (2.1fold) and MHCII (2.4 fold), and downregulation (2.1fold) of B2m were noted.

Of the Th1 cytokine genes investigated, in the non-pregnant immunized mice, IFN-γ was up and downregulated respectively at 24 hrs and 2 weeks post-dose (2.8, 3.1fold) while IL-2 remained downregulated at both time points (7.6, 2.5fold). As compared to 2-4fold downregulation of all the genes in early pregnancy, except for IL-15 (2.3fold), all the other genes were at normal levels post-immunization. As against normal expression of TBX-21/IL-15/IL-12α and downregulation of IL-12β (4fold), IL-2 (4.3fold) and IFN-γ (3fold) during late pregnancy, IL-12α and IL-15 levels were normal and IL-12β (2.6fold), IL-2 (3.23fold), IFN-γ (2.9fold) and Tbx21 (2.3fold) were downregulated at 2 weeks post-dose.

In non-pregnant mice, both the Th2 cytokines (IL-4/IL-10) were downregulated at 24 hours (2.7fold and 2.3fold) and 2 weeks (5.7fold and 3.4fold). At 8 days and 2 weeks of normal pregnancy respectively, unaltered and 4.2fold lower IL4 gene expression respectively was noted while IL-10 was downregulated throughout (2.7fold and 5.9fold). On immunization, IL-4 and IL-10 expression was lower at 8 days (3.7fold and 2.7fold) returning to normal levels at 2 weeks.

Table 3 provides comparisons of serum IL-2/IL-4/IFN- γ levels with the expression of respective genes in the spleen and induction of these cytokines by rNEp-stimulated cultures of splenocytes isolated at the same time point. In the non-pregnant mice, at both points post-immunization, serum cytokines correlated with spleen gene expression. When the splenocyte cultures at 2 weeks post-immunization were stimulated with rNEp, both IL-4 (2fold) and IFN-γ (6.4fold) increased suggesting Th1 bias. In the pregnant, immunized mice, discordance between serum cytokines and gene expression was noted. In non-immunized pregnant mice, a correlation was seen for IL-4 and IFN- γ. At 24 hours post-immunization both non-pregnant and pregnant mice behaved similarly; at 2 weeks, a Th2 bias was evident.

**Table 3:** **Cytokine levels in the sera / supernatants of stimulated-splenocytes and expression of corresponding genes in the spleens of non-pregnant and pregnant immunized mice at different time points**

**Category and time after the dose**	**Serum cytokines (pg/ml)**			**Gene expression spleen (fold change)**			**Splenocytes (rNEp-stimulated , pg/ml)**		
	**IL-2**	**IL-4**	**IFN-γ**	**IL-2**	**IL-4**	**IFN-γ**	**IL-2**	**IL-4**	**IFN-γ**
NPR 24 hrs*	UD	UD	UD	7.6D	2.7D	2.8U	5	5	4.7
NPR 2 weeks	UD	UD	UD	2.5D	5.7D	3.1D	5	10	30
PI 24 hrs**	19.4	UD	56.6	0.7	3.7D	0.9	5	4.5	4.8
PI 2 weeks	2.7	UD	6.2	3.2D	0.9	2.9D	4.5	20	10
PNI day 8***	22.2	UD	UD	2.6D	2.2D	3.2D	4.8	4.5	4.2
PNI day 18	4.7	UD	UD	4.3D	4.2D	3D	5	4.5	4.8

*indicates non-pregnant immunized mice, **indicates pregnant immunized mice, ***indicates pregnant unimmunized mice. UD = undetectable, D = downregulation, U = upregulation.

### Histopathology and biochemical markers

**Figure 7:**
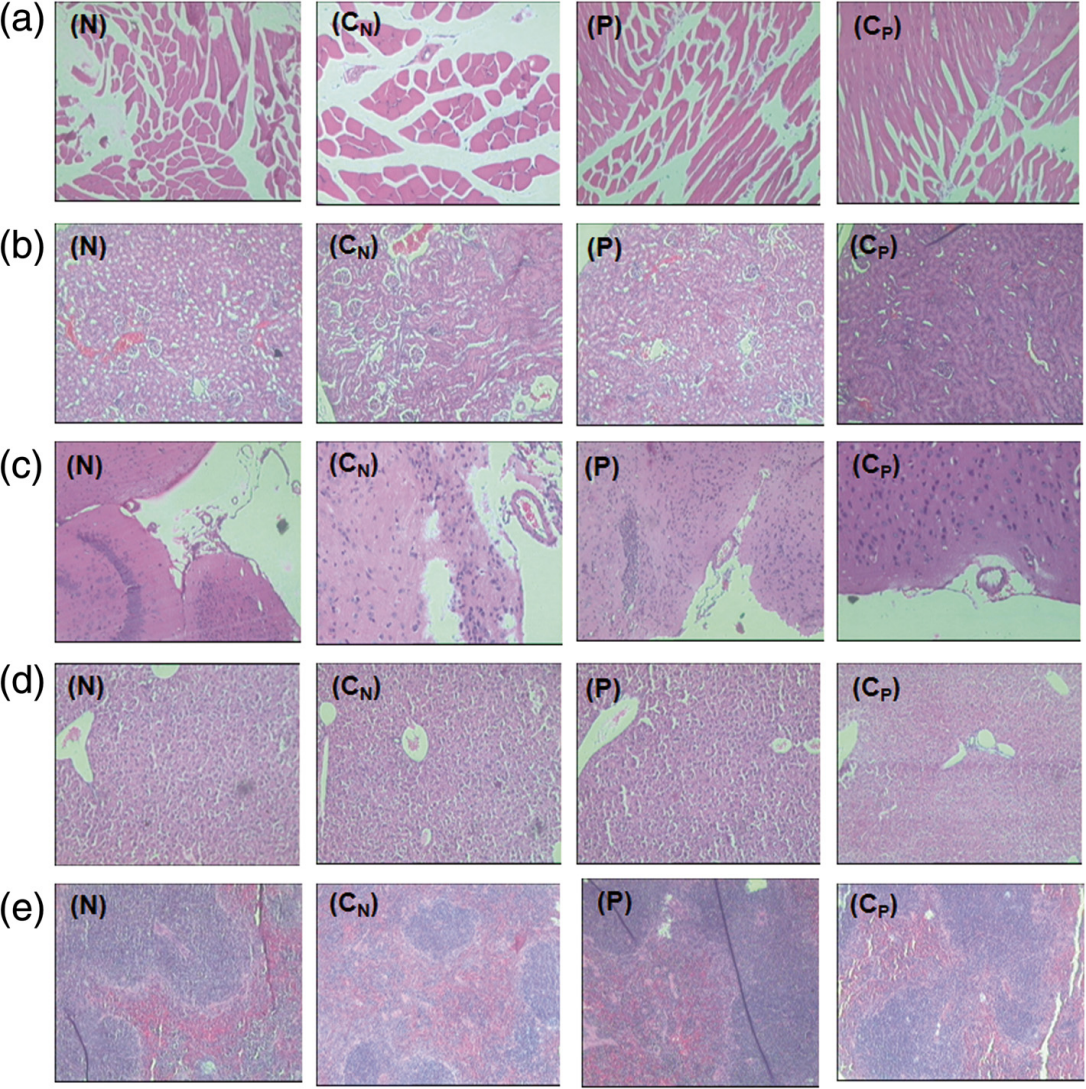
Histopathology findings of **(a)** muscle **(b)** kidney **(c)** brain **(d)** liver and **(e)** spleen in immunized non-pregnant (N) and pregnant mice (P), 24 hrs post dose. C_N_ represents unimmunized non-pregnant mice controls and C_P_ denotes unimmunized pregnant mice controls.

No abnormalities were recorded when tissue pathology (spleen, liver, kidneys, brain and muscles) of the immunized, pregnant/non-pregnant mice and respective non-immunized controls were compared (Figure 7). Levels of SGPT (4-22U/L), SGOT (74-125U/L), urea (40-80 mg/dl) and creatinine (0.2-0.6 mg/dl) were within the normal range for all the groups.

## Discussion

Our vaccine candidate was earlier evaluated in mice (1 μg, 3 doses 1 month apart) [19] and monkeys (20 μg, 2 doses 1 month apart) [8] and shown to be highly efficacious in the rhesus model. As we were planning to give a single dose and pregnancy may hinder antibody response, a higher dose of 10 μg dose was chosen. The results clearly show that even a single dose of the vaccine could induce 100% seroconversion and high anti-HEV titres in both balb/c and C57BL/6 mice, irrespective of pregnancy.

The vaccine was well-tolerated by the pregnant mice immunized during early second para as no obvious adverse symptoms or abnormal biochemical parameters/tissue pathology were noted (Figure 7). Higher anti-HEV titres in both balb/c and C57BL/6 pregnant mice (p < 0.001 for both, Figure 2a, 2b) clearly indicate that the enhanced titres during pregnancy did not reflect genetic makeup of the host, but, driven by the formulation used. In this regard, a report from China is noteworthy [14]. A review of the records of the large phase 3 clinical trial of the vaccine [12], now sold as Hecolin® in China identified that 37/31,791 pregnant women were inadvertently given one (n = 22), two (n = 14) or three (n = 1) doses of the vaccine. Adverse events were comparable in pregnant and non-pregnant women and the vaccine was safe for the mother and fetus. Of note, in the only woman who received 3 doses of the vaccine, antibody level increased from undetectable to 34.7 Wu/mL, which was higher than that of 80% of subjects who received three doses of the vaccine. Overall, results in humans [14] and two mice strains (present study) seem to be comparable and worth pursuing further. Importantly, the 2 vaccines represent 458-607 nt (150aa, this study) and 368-606 nt (239aa, Hecolin®) regions of ORF2.

The other important point to note is that higher antibody response during pregnancy is not a universal phenomenon led by pregnancy-associated Th2-bias but appears to be immunogen-driven. MF59 adjuvanted Focetria H1N1pnd09 influenza vaccine elicited lower antibody titres in pregnant women when compared to the non-pregnant women [20] whereas though statistically insignificant, rabies vaccine induced higher antibody titres in the pregnant women [21]. The observed higher anti-HEV titres in pregnant mice are encouraging for a vaccine intended for use in pregnant women.

To understand the association of increased anti-HEV titres during pregnancy, we determined (1) proportion of T helper (CD4+) and antigen presentation cells (CD11c^+^, CD11b^+^ and CD19^+^) in the splenocytes (2) antigen-specific cytokine induction in the spleen and (3) serum Th1/Th2 cytokine levels. A significant rise in CD11c^+^ dendritic cells in the non-pregnant mice (Figure 4c) suggests involvement of dendritic cells in antigen presentation with the single dose of rNEp-liposome formulation. Similar results were obtained when 2 doses of an HA-based vaccine with Matrix-M were administered to mice [22]. It is interesting to note that when a single dose of ovalbumin was administered with cationic liposomes, though the number of DCs in bone marrow did not increase after immunization, enhanced actin-dependent active endocytosis was noted confirming the role of DCs [23]. Overall, similar to adjuvants like Matrix M [22], MPL-A [24], CpG and LTK63 [25] and cationic liposome DDA [23], rNEp-liposome acts through DCs. In contrast, differential activation of immune cells during pregnancy (Figure 4a, b) was evident by an increased number of CD19^+^ and CD4^+^ splenocytes in the pregnant mice that correlated with higher anti-HEV titres. With attenuated Salmonella vaccine, immunization of non-pregnant mice led to rise in CD8 cells while the numbers in pregnant mice remained unchanged [26].

NE-stimulated splenocytes from the immunized, non-pregnant mice favored Th1 response (Figure 5b). Thus, irrespective of a single dose (10 μg, this study) or 3 doses of 1 μg each [19], the vaccine formulation led to Th1 response. However, the pregnant mice generated a distinct Th2 cytokine response (Figure 5c, d). The levels of IFN-γ were significantly lower in the pregnant immunized mice (Figure 5b). These observations reveal that similar to Salmonella vaccine, pregnancy modulated Th1 response to the vaccine to a Th2 response. A single dose of 1, 5, 10 μg of VLPs of 56 kDa swine HEV-ORF2 with alum induced a Th1 response when splenocytes were stimulated with the same antigen three weeks post intra-muscular immunization [27]. Taken together, these results suggest that irrespective of size of the protein or adjuvant, HEV-ORF2 induces Th1 response in mice and pregnancy dictates a distinct shift to Th2-bias.

Assessment of systemic Th1/Th2 responses (Table 1) revealed absence of Th1 cytokines (IFN-γ, IL-2 and IL-12) and low levels of Th2 cytokines (IL-6 and IL-10) in non-pregnant mice. This cytokine profile is in accordance with the results obtained with a single dose of trivalent influenza vaccine with MF59, alum and other adjuvants [28]. Several studies have noted discordance between Th1/Th2 profiles in spleen and sera in non-pregnant mice [19,29].

During pregnancy, the maternal immune response is altered to accommodate fetus and pregnancy is believed to be associated with a systemic shift towards a Th2 cytokine profile [30]. However, this hypothesis is being debated [31]. With the advancement of pregnancy, the Th1/Th2 (IL-2/IL-10) ratios decreased in both unimmunized (1.46 to 0.18) and immunized (1.71 to 0.12) pregnant categories, suggestive of Th2 bias (Table 2).

Gene expression profile differentiated immunized pregnant mice at 2 weeks from the other groups suggesting vaccine-induced alterations (Figure 6). A concomitant increase in the CD19 gene expression and surface expression of this marker in the pregnant immunized mice is noteworthy. An upregulation of CD40Ig, CD80 and MHCII genes in the pregnant immunized groups suggest their role in generating an enhanced immune response to the formulation. When we compared serum IL-2/IL-4/IFN-γ cytokines and expression of corresponding genes in the spleen (Table 3), a complete correlation was observed in non-pregnant immunized mice and unimmunized pregnant mice (except IL-2). However, discordance was noted in the pregnant immunized group that is under the influence of the pregnancy as well as immunization. As postulated by Keene, probable role of ribonome, an infrastructure between the genome and proteome that may regulate early response genes such as cytokines cannot be ruled out [32].

We further showed that the vertically transferred IgG anti-HEV antibodies could be detected in the pups till 9 weeks after birth (Figure 3a). An earlier report described passive transfer of antibodies after immunizing Swiss albino mice prior to mating with three doses of vaccine formulations [33]. Studies have shown that antibody responses to active immunization with different vaccines vary in the presence of vertically transferred antibodies. In humans, vaccines for hepatitis A [34], measles [35] and rotavirus [36] led to impaired antibody response while no effect was recorded for hepatitis B [37] and Polio [38] vaccines. Reduction in antibody titres for rotavirus in both mice [39] and humans [36] is noteworthy. We showed for the first time that immunization of mice with vertically transferred IgG anti-HEV antibodies resulted in lower antibody response than that observed in age-matched controls without such antibodies (Figure 3b). Importantly, at the time of immunization, the mice were IgG anti-HEV negative. Though hepatitis E vaccine is not envisaged to be administered to the infants from endemic countries, as the disease is seen mainly in young adults, our results will have implications in deciding vaccination age in such countries.

Considering the need for this vaccine in pregnant women, the data generated in mice is very encouraging and must be extended to higher animals. It is also important to evaluate if the higher titres are adjuvant dependent. The results will help in specifically formulating vaccines that will be beneficial for pregnant women, the vulnerable group with high mortality.

## Conclusion

Our study shows that the vaccine candidate is well-tolerated during pregnancy in a murine model and induces high anti-HEV titres. This could probably be associated with the increased CD19^+^ cells along with Th2 cytokines in the spleen and a Th2 bias observed in sera at 2 weeks post immunization. The preliminary data suggests differences in the mechanisms of immune response to the immunogen in pregnant and non-pregnant mice that needs further evaluation.

## Methods

### Immunogen preparation

The recombinant NE protein (rNEp) was cloned, expressed and purified as described previously [18,19]. Immunogen preparation was similar except increase in rNEp to 10 μg. Endotoxin levels were measured using LAL chromogenic endotoxin quantitation kit (Thermoscientific, U.S.A). The cationic liposomes were prepared using cholesterol, hydrogenated soy phosphatidyl choline (HSPC) and stearyl amine. The antigen: liposome mass ratio was 1: 200, w/w). The mixture was lyophilized and reconstituted overnight in sterile PBS before use.

### Studies in mice

The study was approved by the Institutional Animal Ethical Committee and carried out according to the guidelines.

### Immunization schedule and specimen collection

Eight weeks old pregnant and age-matched non-pregnant balb/c (n = 8 for both groups) and C57BL/6 (8 non-pregnant, 5 pregnant) mice were immunized intramuscularly with a single dose (10 μg) of the vaccine candidate. Pregnant mice were administered the dose on day 7 of pregnancy i.e., early second para. Blood samples were collected by retro-orbital bleeding prior to and one and ~ two weeks post-dose and sera was stored at −20°C/-80°C till tested for anti-HEV IgG titres and cytokines. In C57BL/6 mice, only antibody response was studied.

**Figure 8:**
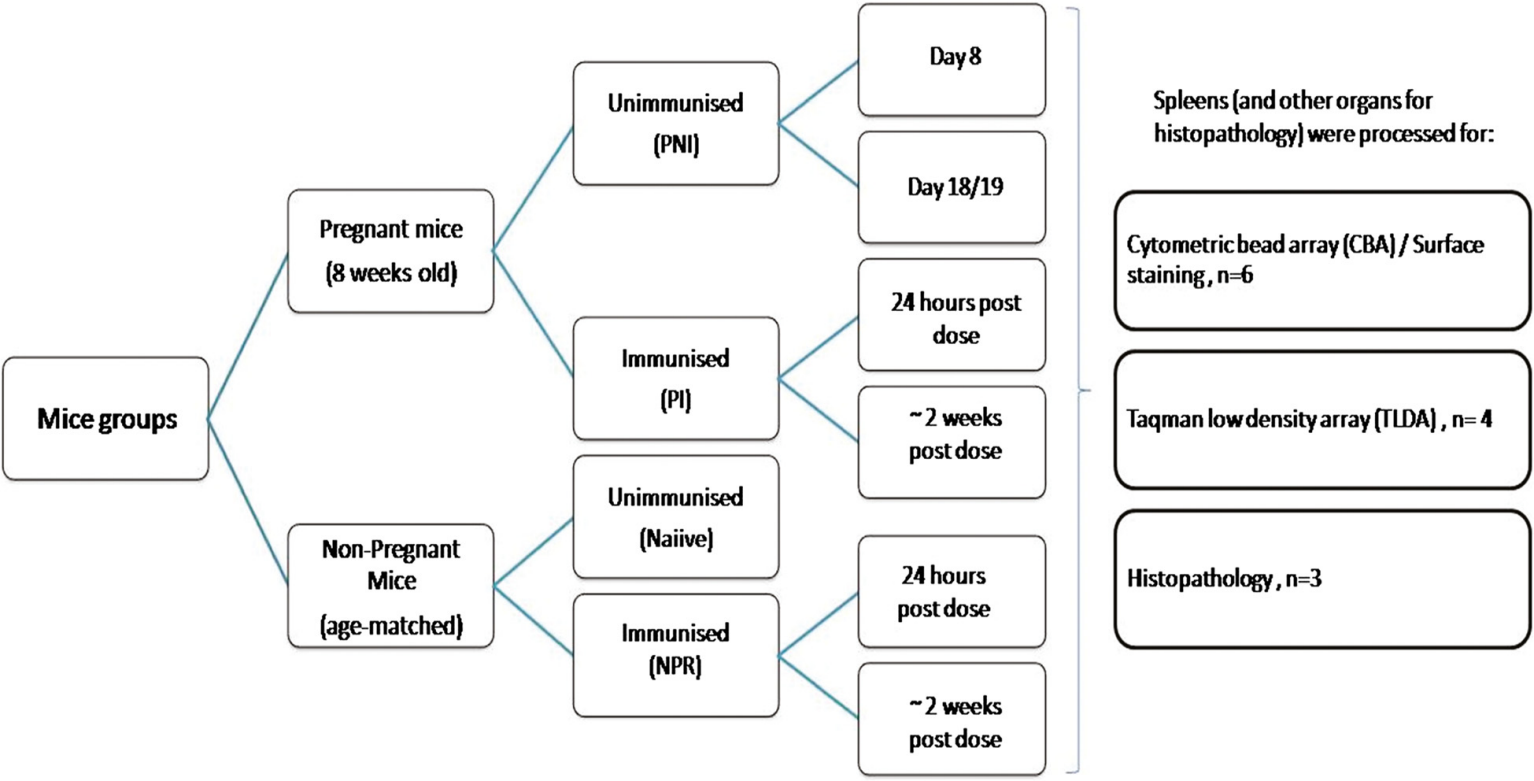
Study protocol describing various mice groups and numbers used for organ harvesting.

Both non-pregnant (NPR) and pregnant immunized (PI) mice were sacrificed at (1) 24 hours-post-dose and (2) ~ two weeks-post dose. As controls, unimmunized pregnant mice (PNI) were sacrificed at the same time points. Unimmunized non-pregnant (naiive) mice served as controls (Figure 8). The spleens were harvested and used for surface staining, Cytometric Bead Array (CBA) and Taqman Low Density Array (TLDA).

### Vertical transfer of anti-HEV antibodies and response to active immunization

To assess the persistence of vertically transferred IgG anti-HEV antibodies, 8 pregnant, immunized mice were kept for delivery and a total of 41 pups were born. The pups were bled at regular intervals for 14 weeks. To determine the effect of passively transferred antibodies on immune response to active immunization, in a separate experiment, set of pups (n = 10) born to different vaccinated mice were immunized at the age of 13 weeks. Age matched mice born to non-immunized, anti-HEV negative mothers (n = 10) were used as controls.

### ELISA and serum cytokines

Anti-HEV positivity and titres were determined by rORF-2 based ELISA [18]. Serum cytokines were measured by milliplex mouse cytokine kit (Millipore, Germany). Samples from the same group were pooled and run in triplicates.

### Immunophenotyping of splenocytes

Single cell suspension of splenocytes was prepared as per standard protocol in RPMI by teasing the spleen followed by R.B.C lysis (BD Pharmlyse) and passing through a 70 μ cell strainer (BD Biosciences) .The anti-mouse antibodies used for staining were: T-cell markers: FITC-CD3 (clone17A2)/PE-CD4 (GK1.5)/B cell marker: APC-Cy7-CD19 (ID3)/macrophage marker: FITC-CD11b (M1/70)/Dendritic cell marker: APC-CD11c (HL3) (BD Pharminogen, U.S.A). Splenocyte cluster was gated and a minimum of 20000 events were acquired for every sample on BD FACS ARIA II flow cytometer. Samples were analyzed using BD FACS DIVA software. (BD Biosciences, U.S.A).

### Cytometric Bead array (CBA)

To assess the Th1/Th2 cytokines in the supernatants of splenocytes, CBA kit (BD Biosciences, U.S.A) was used as described previously [40]. Briefly, 1 million cells were seeded in RPMI supplemented with 2% FBS per well and were stimulated with 1 μg of rNEp. Cell supernatants were collected after 72 hrs and stored in −80°C till tested. Concanavalin A stimulated (2.5 μg/well) and unstimulated cells served as positive and negative controls respectively. Values represent mean cytokine measurements of individual mice of a group ± Standard error (SE).

### Taqman Low density Array (TLDA)

Total RNA extraction and gene expression analysis was done from frozen spleen samples as described previously [41]. The TLDA card was loaded on to the 7900HT Fast Real Time PCR system (Applied Biosystems). 500 ng of RNA was used for cDNA synthesis cDNAs from spleens of naive mice were used as calibrators.18 s gene was kept as endogenous control. Relative quantification (RQ) values below 0.5 were considered as downregulation and values greater than 2 were considered as upregulation.

### Histopathology and serum biochemical markers

Spleen, liver, kidney, brain and muscles at the site of injection were harvested, stored in 10% buffered formalin and processed for histopathology as per standard protocols. Serum liver transaminases (SGPT and SGOT), urea and creatinine levels were determined for all the mice groups using Dimension RXL Max machine (Siemens, Germany).

### Statistical analysis

Student’s t test and ANOVA were performed using SPSS 11 software. Non-responders in each group were included for analysis.
